# Exploring the actionability of healthcare performance indicators for quality of care: a qualitative analysis of the literature, expert opinion and user experience

**DOI:** 10.1136/bmjqs-2020-011247

**Published:** 2021-05-07

**Authors:** Erica Barbazza, Niek S Klazinga, Dionne S Kringos

**Affiliations:** Department of Public and Occupational Health, Academic Medical Centre, Amsterdam University Medical Centres, University of Amsterdam, Amsterdam, The Netherlands

**Keywords:** quality measurement, health services research, healthcare quality improvement, management, performance measures

## Abstract

**Background:**

This study explores the meaning of actionable healthcare performance indicators for quality of care-related decisions. To do so, we analyse the constructs of *fitness for purpose* and *fitness for use* across healthcare systems and in practice based on the literature, expert opinion and user experience.

**Methods:**

A multiphase qualitative study was undertaken. Phases included a literature review, a first round of one-on-one interviews with a panel of academics and thought leaders in the field (n=16), and a second round of interviews with real-world users of performance indicators (n=16). Thematic analysis was conducted between phases in order to triangulate findings in a stepwise process.

**Results:**

Common uses of healthcare performance indicators were differentiated within micro-meso-macro contexts of healthcare systems. Each purpose of use signals different decision-making tasks, and in effect information needs. An indicator’s fitness for use can be appraised by three clusters of considerations: methodological, contextual and managerial. Methodological considerations gauge an indicator’s perceived importance, engagement potential, interpretability, standardisation, feasibility of remedial actions, alignment to care models and sensitivity to change. Information infrastructure, system governance, workforce capacity and learning culture were found as enabling contextual considerations. Managerial considerations influencing an indicator’s use in practice were found to span the selection of indicators, data collection, analysis, display of results and delivery of information to decision-makers.

**Conclusion:**

The actionability of a healthcare performance indicator should be appraised by its alignment with the intended purpose of use beyond aggregate healthcare system levels, in combination with the extent to which methodological, contextual and managerial fitness for use considerations are met. Striking a better balance between the importance weighted to an indicator’s statistical merits and emphasis put to its fitness for purpose and use is needed for indicators that are ultimately actionable for quality of care-related decision-making.

## Introduction

Healthcare performance measurement, and its use as performance intelligence, plays an important role in guiding the decisions of healthcare system actors with respect to quality of care.^1^ Since the early 2000s, the importance of performance measurement in healthcare,^2^ its institutionalisation as standard practice within^3^ and across healthcare systems,^4–6^ and more recently its professionalisation^7^ has received widespread prioritisation. This attention has increased scientific rigour around criteria for selecting indicators (eg, reliability, validity),^8 9^ development of indicator sets (eg, parsimony, epidemiological relevance),^10^ and methods, tools and approaches to guide these processes.^11–13^

Importantly, adherence to agreed-upon criteria for a statistically sound indicator does not guarantee that it is useful for decision-making. The information needs of decision-makers across healthcare systems, including policy-makers, managers, clinicians and patients, are varied. The type of indicator, data sources, level of precision, timeliness and relevant comparisons are among the key differences.^1 14 15^ For example, working to improve antibiotic prescribing, a primary care clinician may assess new and represcribing of antibiotics in their practice quarterly; an insurer, the adherence of practices to prescribing guidelines for issuing payment incentives annually; and a policy-maker, the total volume of antibiotics prescribed per 100 000 population by region, nationally and in comparison with other countries by policy cycle.

In effect, the ability for an indicator to meet the information needs of decision-makers goes beyond their statistical quality and is rather a measure of their actionability. To be actionable, it is generally agreed an indicator should be both *fit for purpose*—serving an intended decision-making function—and *fit for use*—getting the right information into the right hands at the right time.^16–18^ While there is agreement on the importance of actionability,^18–20^ and increasing attention put to its two main constructs of fitness for purpose and use, it still remains an elusive concept to define, assess and operationalise. In the absence of a common understanding of the meaning of actionability, the tendency to select indicators on the merit of their *potential* to be actionable perpetuates.^18 21–23^ And while there are implicit criteria that appear to influence the actual use of indicators, such as data availability and ease of interpretation,^1 15 24–26^ how these relate across different healthcare systems remains underexplored.^1 14 15^

With the advancement of information systems and data analytics, there has been impressive growth in the speed, volume and range of data available for performance measurement.^27 28^ COVID-19 and the ensuing surge in performance data reported is evidence of this.^29 30^ It also serves to illustrate that an abundance of information does not translate to informed decisions. Our attention is increasingly called to this fact and the work still needed to advance methods for measuring quality of care^31–33^ and patient safety^34^ in order to obtain additional value from our data-rich systems.^35–38^

In this study, we set out with the aim to gain further insights into the meaning of actionable healthcare performance indicators for quality of care-related decision-making across healthcare systems. To do so, we explore the notions of fitness for purpose and fitness for use derived through the existing literature, expert opinion and experiences of data users in varied developed country contexts. We pose two questions. The first aims to differentiate an indicator’s purpose of use by micro-meso-macro decision-making levels, investigating what are the uses of healthcare performance indicators across healthcare systems. The second aims to consolidate the determinants of an indicator’s fitness for use, exploring what are the key considerations influencing an indicator’s use.

## Methods

### Design

We applied qualitative methods^39^ in a multiphase approach, comprising a review to examine actionability according to the published literature^40^ and multiple perspective semistructured interviews^39 41 42^ to gain insights from two groups (panels) representing the scientific community and data users. We employed one-on-one interviews following our literature review rather than a questionnaire or focus groups for richer exchanges and the possibility to elicit the individual opinions of each participant.^43^ Our stepwise approach to analysis allowed for the triangulation of findings across phases and to aggregate individual-level results for panel-wide themes.^42^ The study adheres to the Consolidated Criteria for Reporting Qualitative Research.^39^

*Indicators* refer to a quantifiable variable measured to provide simplified information about a larger area of interest,^44^ typically measured over time.^9 45^ In the scope of this study, we focus on *healthcare performance indicators*: indicators for quality of care-driven decision-making to improve performance on one or more of the six dimensions of quality: safe, effective, patient-centred, timely, efficient and equitable care.^8 46^ As an exploratory study, we prioritised the generalisability of findings and were inclusive of varied types of healthcare (eg, primary, acute, specialist, long-term care), settings (eg, primary care, hospitals), health system types and countries, although limited to developed country contexts.

**Figure 1:**
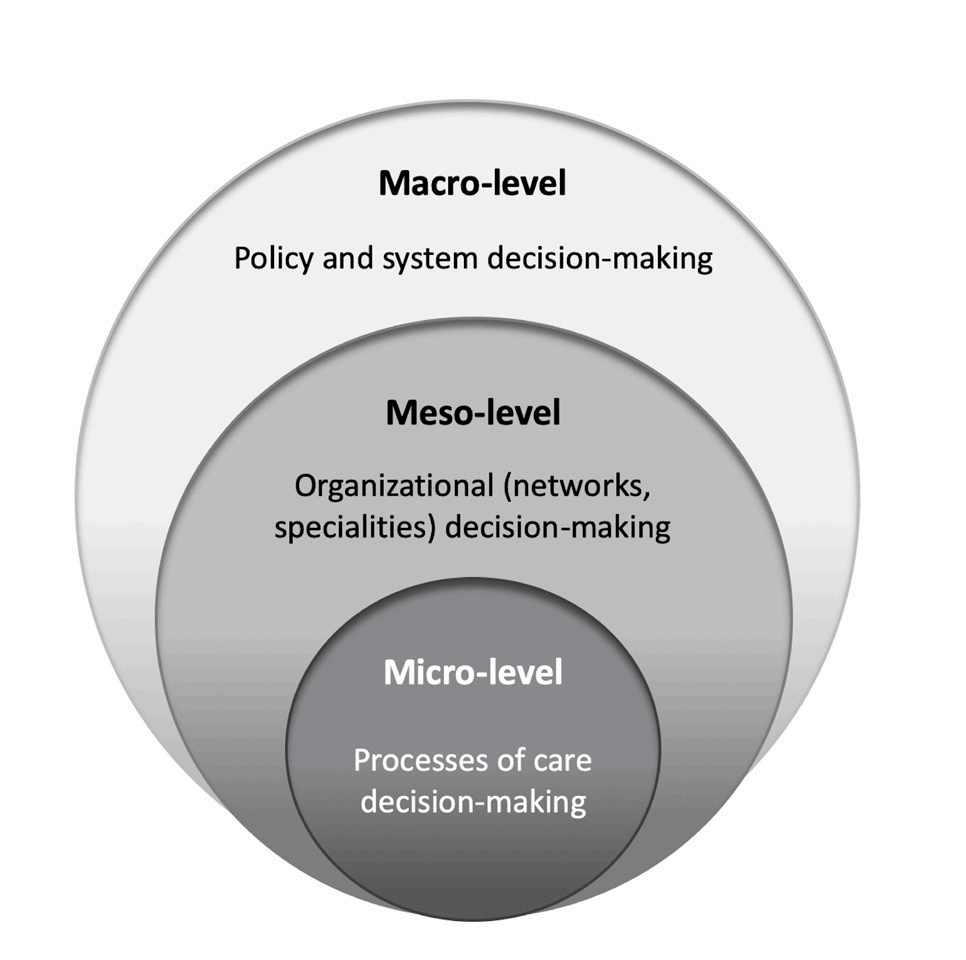
Decision-making contexts across healthcare systems.

To explore our first research question, we took as a basis the characterisation of decision-making in healthcare systems by three contexts: patient care (micro-level), organisational (meso-level) and policy (macro-level), as illustrated in figure 1.^47 48^ Indicators are used to inform decisions in each context, be it quality improvement, services management, population health planning or other strategic and tactical choices.

### Data collection and analysis

#### Phase 1: literature review and content analysis

We reviewed the existing literature with the following aims: to examine the current scientific understanding of actionable healthcare performance indicators; to generate an initial core list of indicator purposes of use and fitness for use considerations; and to identify leading experts in the field. Our search was conducted using PubMed at the outset of the study in early 2019. The search was limited to the past 10 years and articles published in English using the following key terms in varied combinations: health care performance indicator, actionability, quality of care, measurement and use. We also reviewed reporting of relevant international organisations and networks, namely the WHO and its regional offices, the Organisation for Economic Co-operation and Development (OECD), and the European Commission Expert Group on Health Systems Performance Assessment. Reference lists of articles and reports identified were reviewed in a snowballing approach.

The results of the initial literature search were synthesised and used to inform a provisional approach and visualisation of the uses of healthcare performance indicators by micro-meso-macro context. Recurrent fitness for use considerations were also distilled and clustered. These findings were prepared as an expert panel brief for use as a background document in the second phase (online supplemental appendix 1).

10.1136/bmjqs-2020-011247.supp1 Supplementary data

#### Phase 2: interviews with expert panel and thematic analysis

The first panel aimed to engage prominent academics and thought leaders in the field of healthcare performance measurement and quality of care (hereafter, expert panel). Experts were identified based on the authorship of literature reviewed and with consideration to the following criteria for the panel’s composition: a balance of expertise in areas related to quality of care, performance measurement, governance, data and information systems or management; senior academic or technical roles related to their area of expertise; and affiliation to varied healthcare systems and geographical contexts. A target of 15 experts were pursued for manageability and presumed saturation.^49^

One pilot interview was conducted to ensure relevance and clarity. Piloting resulted in the addition of illustrative examples of data users and fitness for use considerations. Panellists were invited to participate via email and received a panel brief in advance. The brief provided relevant study details together with the findings of phase 1. All interviews were conducted by the primary researcher (EB, female) with experience in semistructured interviews and subject matter expertise. Interviews took place between August and September 2019 both in person and at distance based on the proximity and preference of panellists. Interviews lasted between 45 and 60 min. Records of the interviews were prepared as detailed summaries rather than verbatim transcripts in the approach described by Halcomb and Davidson.^50^ The research adheres to the Dutch ethics guidelines stated in the ‘Medical Research Act with People (Wet medisch-wetenschappelijk onderzoek met mensen (WMO)) (Dutch), in BWBR0009408, W.a.S. Ministry of Health, Editor. 1998: Hague, Netherlands’,^51^ for which verbal consent was deemed adequate by the authors as no human data were retained. To ensure informed voluntary participation, participants provided written agreement to participate in the study during the recruitment stage and restated verbally their consent at the start of all interviews.

The interview records of this first panel were stored in an Excel-based tool for thematically analysing themes (EB). The analysis incorporated a deductive and inductive approach: topics explored in the interviews (online supplemental appendix 1) were used to guide the deductive thematic analysis^52^ and new themes that emerged were identified using an inductive approach.^53^ The data were also interpreted by redrawing conceptual diagrams. Two others (DSK, NSK) with complementary expertise in quality of care, performance measurement, health governance and management reviewed the findings to ensure consistency and reach agreement on the theme extraction.

#### Phase 3: interviews with user panel and thematic analysis

The findings from the expert panel were used to refine the mapping of uses of healthcare performance by micro-meso-macro level and fitness for use themes. The revisions were summarised in a new brief prepared for a second panel of one-on-one interviews (online supplemental appendix 2). This panel aimed to engage real-world data users for their first-hand experiences using healthcare performance indicators for quality of care-related decision-making (hereafter, user panel).

A target of 15 data users actively contributing to the further development of this field were pursued as panellists. The selection drew on existing membership lists of international networks, working groups and projects related to healthcare performance indicators, measurement and quality of care, such as the OECD Health Care Quality Indicators Project^54^ and initiatives of the European Commission (eg, HealthPros^55^). The panel composition aimed to capture a range of perspectives, with representation of differing health system types, country affiliations and uses of healthcare performance indicators. Interviews were conducted in the same manner as the first panel and were completed between November 2019 and January 2020.

Interview records were consolidated in the existing Excel-based tool for further thematic analysis. The topics and themes explored were used to refine and/or confirm the classification resulting from the expert panel on uses of healthcare performance indicators and fitness for use considerations. Observing the convergence of themes, with this phase data collection and analysis were considered complete.

## Results

### Literature review and panel results

Based on the literature synthesis, 19 experts were identified and invited to participate in the first panel. Of these, 16 experts agreed to participate. Non-participants were either unreachable (n=1), unavailable (n=1) or referred to an alternative contact (n=1). Together, expert panellists had published more than 50 articles or reports on the use, selection or improvement of healthcare performance indicators at the time of study. This literature (online supplemental appendix 3) was reviewed in phase 1 together with other relevant works.^22 34 44 48 56–64^ Expert panellists were predominately affiliated to academia and in senior or executive roles spanning eight countries (Australia, Canada, Denmark, Germany, Italy, the Netherlands, UK and USA). A range and balance of areas of expertise that included performance measurement, quality of care, governance, information systems and management were achieved.

The user panel comprised participants spanning the micro-level, meso-level and macro-level of healthcare systems. Participants included representatives of national health authorities, health standards and accreditation agencies, insurers, professional associations, as well as clinicians and patient advocates. In total, 31 participants were contacted, of which 16 agreed to participate (online supplemental appendix 3). Non-participants reported the same reasons as the first panel, with the majority (n=6) referring to an alternative contact and the remainder being either unreachable (n=5) or unavailable (n=4). User panellists spanned seven countries (Belgium, Canada, Germany, Ireland, the Netherlands, UK and USA). Table 1 summarises the key characteristics across panellists.

**Table 1:** Characteristics of panellists

Expert panel	n (%)	User panel	n (%)
Total	16 (–)		16 (–)
Affiliation*		Uses	
Academia	10 (63)	Macro	7 (44)
International organisation	3 (19)	Meso	4 (25)
Think tank	3 (19)	Micro	3 (19)
Expertise		Organisation type	
Measurement	5 (31)	Government	5 (31)
Quality of care	3 (19)	Health services	4 (25)
Governance	3 (19)	Standards	3 (19)
Information systems	3 (19)	Research	2 (13)
Management	2 (13)	Improvement	2 (13)
Region			
Europe	9 (56)		9 (56)
North America	5 (31)		7 (44)
Oceania	2 (13)		–
Sex			
Male	11 (69)		9 (56)
Female	5 (31)		7 (44)

*Primary affiliations.

**Figure 2:**
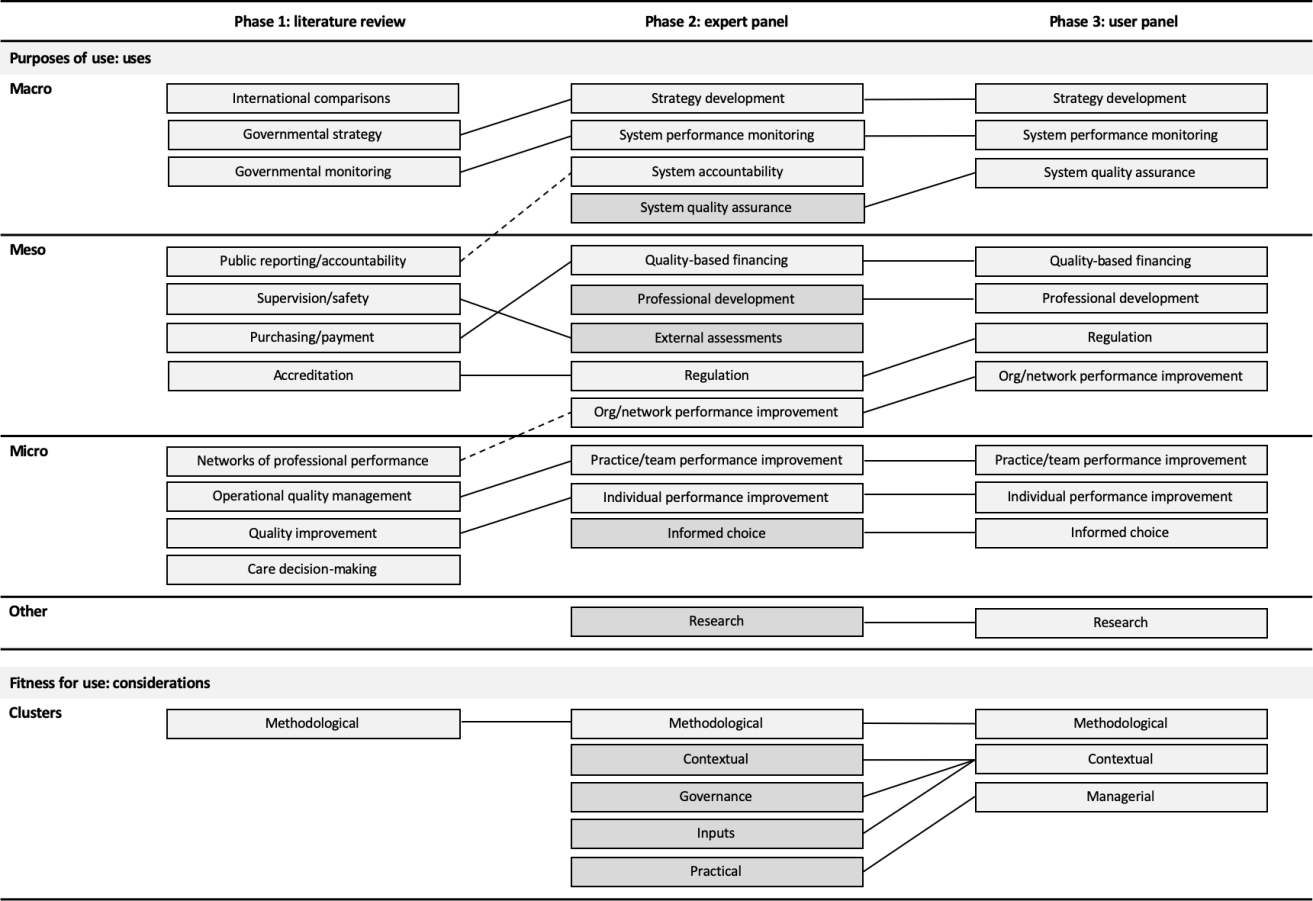
Summary of key findings across study phases. Note: boxes denote key themes emerging by study phase. Broken lines denote a change in level. Solid lines denote agreement between phases with possible adjustments to phrasing. Darker grey shading denotes the introduction of new elements. Ordering within cells is not indicative of importance.

From the literature reviewed, 11 clusters of uses of healthcare performance indicators and fitness for use considerations related to the methodological quality of an indicator were identified (figure 2). In the second phase, there was agreement across experts on the relevance and importance to distinguish purposes of use of healthcare performance indicators beyond aggregate micro-level, meso-level and macro-level. The panel shared strong views to avoid a hierarchy within levels, finding this introduced a rigidity that may not translate across contexts. Rather, the framing of uses identified as common or frequent was found more transferable.

The experts introduced further consistency, refinements and additional purposes of use and fitness for use considerations. Specifically, the uses of indicators for functions such as regulation or strategy development were differentiated from mechanisms to achieve these functions, such as international comparisons or public reporting. Refinements to the distribution of uses across levels were introduced for consistency, for example, recategorising *improvements of organisations and networks* to the meso-level. Additions included emphasis on the use of indicators by patients as a decision-maker for informed choice and the cross-cutting function of research. The clustering of fitness for use considerations was disaggregated, with emphasis on the importance of considering an indicator’s use in a specific setting (where it is used) and as a process (how it is used).

In the third phase, user panellists agreed with the categorisation of uses by micro-level, meso-level and macro-level. *Accountability* was viewed as an aim rather than specific use and *external assessments* were viewed rather as a mechanism. There were detailed discussions on fitness for use considerations, with agreement to classify considerations that underscored the importance of the setting in which an indicator is used for its contextualisation. The case was made to view *practical* considerations as *managerial* aspects related to the process of using indicators.

### Purposes of use of healthcare performance indicators

Through our stepwise approach to data collection and analysis, common uses of healthcare performance indicators were differentiated beyond the aggregate decision-making contexts of patient care (micro-level), organisations (meso-level) and policy (macro-level). In table 2, we list the uses for healthcare performance indicators identified, each serving different managerial decision-making functions, users and information needs. The purposes of use are not exhaustive and may take varied forms by healthcare system. Specifically, expert and user panellists noted variation in the degree of patient choice, role of insurers or mandate of professional bodies.

**Table 2:** Differentiating uses of healthcare performance indicators across healthcare systems

Context	Purpose of use	Illustrative uses	Illustrative users	Illustrative information need
Macro	System performance monitoring.	Signalling the performance of the system as a whole; comparing performance internationally; publicly reporting system performance.	Public; ministry of health; regional (provincial, state) authorities; health service executive (authority).	How is my healthcare system doing? How does it compare with others?
	Strategy development.	Setting health policy priorities; identifying emerging health priority areas; and monitoring trends in current priority areas.	Government and ministries; regional (provincial, state) authorities; accountable care organisations; health maintenance organisations.	Have I chosen the right areas to prioritise? What is the impact of strategies that are in place?
	System quality assurance.	Measuring care processes; reporting of incidents and never events.	Quality inspectorate; national quality observatory; health and safety executive.	Is care being delivered as intended? Where do problems in the delivery of care lie?
Meso	Regulation (professional, facility, pharmaceuticals).	Informing accreditation, certification and/or licensing processes.	Medical councils, chambers, college of physicians; medicines and healthcare products regulatory agency.	Does the performance of organisations, facilities, medicines, etc, meet established standards?
	Professional development.	Reporting internally and benchmarking within profession or specialty.	Societies of medical professionals; professional associations; training institutions.	How do healthcare professionals of a specific specialty perform?
	Quality-based financing.	Issuing performance-based payment (pay-for-performance); value-based contracting.	Healthcare insurers; healthcare providers.	Are existing guidelines or standards being adhered to? Does this merit the issuing of incentives?
	Organisation/network performance improvement.	Improving performance of hospitals, networks and care groups; assessing local needs and geographical differences.	Hospital management; integrated care; networks/groups; local collaboratives of care.	Are affiliated practices/facilities performing optimally?
Micro	Practice or team performance improvement.	Convening audit and feedback, plan-do-study-act, and/or collaborative, team-based improvement cycles; comparing across practices.	Primary care practices; specialist departments or units; pathways of care.	How is my team performing? How can we improve our performance? How do I perform relative to my team members?
	Individual performance improvement.	Identifying trends in the management of patients; tailoring services to target groups.	Individual physicians; nurse/practitioners; other healthcare professionals.	How am I managing my practice panel? How can I improve my performance?
	Informed choice.	Selecting a healthcare provider; participating in care decision-making; self-managing care needs.	Patients; family members and carers; public.	What treatment options or providers are best for me?
Cross-cutting	Research.	Exploring the use of indicators across contexts.	Academia and academic networks; think tanks, research groups; topic-specific associations.	Secondary user-directed.

The detailed differentiation of uses of healthcare performance indicators signals important, yet often overlooked, distinctions in information needs *within* system levels. To illustrate these differences, we take the macro-level as an example. While uses of healthcare performance indicators in this context share an overall aim of informing policy decisions, distinctions between uses include system performance monitoring—signalling to system stakeholders, often including the public, the performance of the system as a whole, answering ‘How is my health care system doing?’; or strategy development—signalling to ministries, departments of health or similar with the aim of identifying priority areas, monitor trends and ultimately answering ‘Have I chosen the right areas to prioritize?’; or system quality assurance—informing decisions of health service executives, quality inspectors or quality observatories for an overview of care processes and signalling of incidents, answering ‘Is care being delivered as intended?’

### Fitness for use of healthcare performance indicators

Three main clusters of considerations influencing the second construct of actionability—fitness for use—were found. These include methodological, contextual and managerial considerations (table 3).

**Table 3:** Overview of methodological, contextual and managerial fitness for use considerations

Clusters	Considerations	Guiding questions for considering an indicator’s use
*Methodological*		
	Measures what matters.	Does anybody care?
	Wide engagement.	What can *we* do?
	Easily interpreted.	Does the indicator signal a clear direction?
	Clear standardisation.	Is the indicator clearly defined and replicable?
	Alignment of accountability.	Are entry points for taking action feasible?
	Measurement matches delivery.	Is the indicator a reflection of the system?
	Sensitive to meaningful change.	Is the indicator sufficiently sensitive to change?
*Contextual*		
Information infrastructure	Interoperability.	Can needed data be accessed?
	Data quality.	Is the data of quality?
Governance	Political will and vision.	Is there high-level commitment and direction for use?
	Regulation for data protection.	Does existing legislation facilitate use?
	Cross-sector partnerships.	Are cross-sector partnerships in place?
	Aligned financing structures.	Do financing structures encourage the intended use?
Workforce capacity	Data and quality expertise.	Are the competencies to interpret and use data in place?
	Time dedicated to improvement.	Is time allocated to encourage use?
Culture	Learning orientation.	Is an environment for learning cultivated?
	Shared responsibility for health.	Do users feel accountable for improvement?
*Managerial*		
Selecting healthcare performance indicators	Clear purpose of use.	What is the purpose of use? (eg, strategy development)
	Target end user is known.	Is the target audience known? (eg, clinicians, public)
	Conceptual framework.	Is the dimension of quality pursued clear?
	Indicator quality.	Is the indicator scientifically sound?
	Source, type and availability of data.	What data are needed and are they available? (eg, administrative, clinical, survey data, wearables)
	Standards for appraisal.	How will improvements in performance be assessed?
	Degree of public disclosure.	Is the indicator for internal or external (public) use?
	Accompanying indicators.	Are there relevant accompanied indicators?
	Previous use.	Has the indicator been used previously?
Accessing data	Representativeness of data.	Are the data complete?
	Data linkages.	Can relevant data sources be linked?
	Data collection tools.	How will data be collected? (eg, paper-based, automated electronically, manual electronic entry)
	Unity of language/coding.	Is there consistency in coding across data to be used?
Applying methods of analysis	Type of analysis.	How will the data be analysed? (eg, benchmarking, time trend, case mix correction)
	Aggregation of indicators.	How can composites/indices be used to simplify data?
	Reference group.	Who is the reference group?
	Breakdowns/cohorts.	How will the data be disaggregated? (eg, age, sex, ethnicity, geographically)
	Calculation of values.	How will values be calculated? (eg, mean, median, SD, top 10% mean)
	Time interval.	Should a time trend be reported and at what interval?
	Application of risk adjustments.	How will risk adjustments be applied? (eg, variable specification, source, weighting scheme)
	Managing missing data.	How will missed data points be handled?
	Contextualising data.	What other data are needed to give the indicator meaning?
Displaying findings	Chart options.	How will the data be visualised? (eg, chart, map, table)
	Simplification techniques.	What techniques to simplify the meaning can be applied? (eg, colour, size variation, icons)
	Customisation of display.	How can users customise the data? (eg, change of display, change of information)
	Narrated interpretation.	How can the quality and the meaning of data be narrated?
	Format of reporting.	How will it be reported? (eg, print, mobile, web-based)
Reaching decision-makers	Frequency of reporting.	What is the relevant reporting cycle (eg, real time, quarterly, annually, biennially)
	Dissemination channels.	How will users be reached? (eg, mail, email, champions)
	Guidance on use.	How can users be supported to make use of findings?

#### Methodological considerations

Methodological considerations pertain to the indicator itself, although beyond its statistical quality. Seven recurrent considerations were identified. First, an indicator should measure what matters. User panellists emphasised the importance that the target audience *cares* about the results, explaining an indicator that ‘moves’ people makes everyone uncomfortable that the right thing is not already being done. Second, the extent to which an indicator resonates with a range of stakeholders was emphasised as a key gauge of its ability to facilitate a ‘what can *we* do’ approach, rather than limiting action to an individual user.^65^ Third, an indicator’s inherent ease of interpretation was described by panellists and in the literature^18 66 67^ to strongly influence an end user’s confidence in their interpretation of its meaning. Fourth, the extent to which an indicator is clearly defined was described as a key contributor to trust in what it signals, as well as the likelihood of wide uptake. Fifth, an indicator should be able to be broken down into its constituent parts to make change points clear,^8^ with panellists finding a remote or disconnected indicator from a user’s performance difficult to act on.^59 63^ Sixth, an indicator should measure a phenomenon as true to lived experience as possible.^27 68^ The tendency to focus on specific (siloed) areas of care was described to reduce performance to overly narrow aspects of care and, as one user panellist described, misses the ‘system-ness’ of quality. Lastly, the ability of an indicator to be sufficiently sensitive to change based on its intended use was described by both panels as intuitive, yet often a challenge for an indicator to meet.

#### Contextual considerations

Contextual considerations refer to critical factors pertaining to the setting in which an indicator is used. Four main clusters emerged. One, the information infrastructure was met with consensus across panellists as a key predictor of use, determining the ability to collect, store and extract information. Relevant considerations repeatedly raised included the interoperability of information systems (ie, linkages, output format) and overall data quality (ie, consistency in field, codes, maintenance). Second, characteristics of governance were emphasised, with panellists citing the importance of political will and vision, regulatory arrangements for data exchanges, as well as cross-sector partnerships and aligned financing structures. Third, workforce capacity considerations were underscored, specifically the data literacy skills of actors across the healthcare system and the availability of protected time for the healthcare workforce to use data. Lastly, pertaining to culture and professional norms, be it in clinical practice, healthcare organisations, professional networks or government agencies, the importance of a learning orientation and shared sense of responsibility was emphasised as a predictor of the importance placed to measurement and ultimately the use of an indicator.

#### Managerial considerations

**Figure 3:**
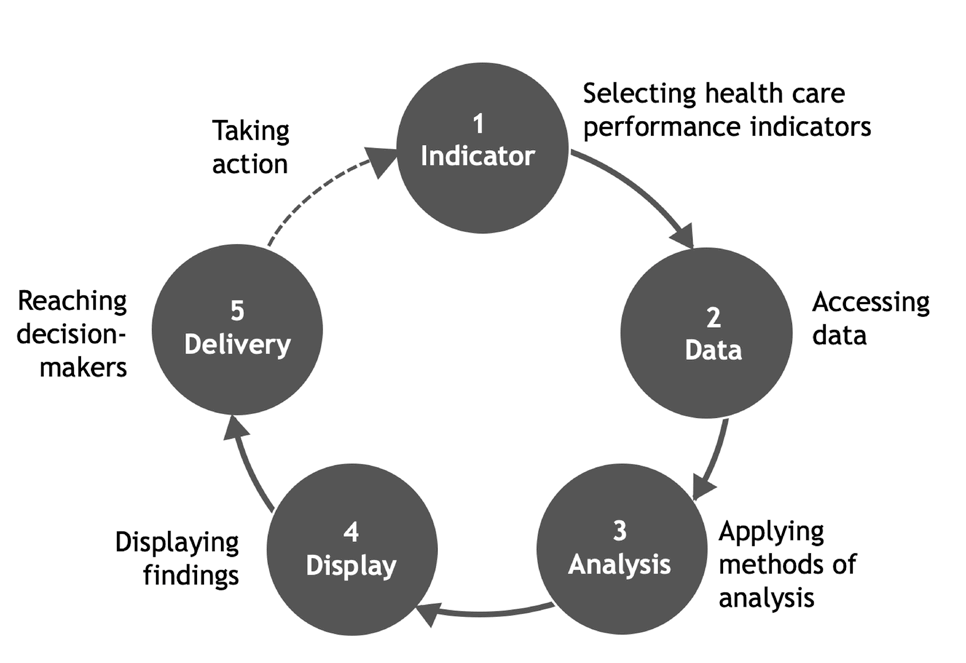
Use cycle for managing healthcare performance indicators.

The importance of embedding indicators into performance management systems is well established.^60 63 69–72^ Based on the literature and insights from the panels, we conceptualised an indicator’s use cycle (figure 3). This cycle was used to consolidate considerations brought forward around embedding indicators into management systems to safeguard an indicator’s use in practice. The considerations reflect key decisions to be managed across the cycle and include selecting an indicator with consideration to define clear parameters of its intended use,^18 38 73^ gain clarity around its construction,^60^ assess data needs and define measurement considerations; accessing data to ensure data are available, of quality or can feasibly be collected^48^; applying methods of analysis for the relevant calculation of values that correspond to the intended purpose^63^; displaying findings, including decisions around how data are visualised^74^ and the degree of *story-telling* to describe and interpret results to support understanding of what is meant and any caveats^48 75 76^; and actually reaching decision-makers, with decisions needed as to the frequency of dissemination, channel used for delivering information and guidance (if any) to facilitate the use of information provided.^63^

## Discussion

### Principal findings

Healthcare performance indicators share a common aim to provide simplified, readily understood information to facilitate decision-making.^9 44 45^ An indicator’s ability to do so in practice extends beyond its statistical quality and rather is characterised by its actionability.^16–18 67^ In this study, we explored actionability through the two constructs of fitness for purpose and fitness for use and observe the following main findings into their further operationalisation.

First, the different uses of an indicator within micro-meso-macro and research contexts stress the importance of clarity and precision on the intended use of an indicator. The relevance of precision regarding an indicator’s use has been stressed in the literature^15 18–20 23^ and previously explored from the perspective of different end users.^1^ Our findings further differentiate uses of indicators across healthcare systems. While not pursuing a universal, exhaustive listing of purposes of use—recognising varied healthcare system types and contextual considerations that deem this irrelevant—our findings signal the imperative of clarity regarding an indicator’s intended use and user to gauge its potential usefulness. The taxonomy of uses of healthcare performance indicators can be an input to further operationalise the construct of fitness for purpose.

Second, we find an indicator’s fitness for use is captured by three types of considerations. These relate to an indicator’s technical qualities, its intended context of use and its handling across what can be characterised as a use cycle. It means, to gauge an indicator’s fitness for use, a range of considerations should be assessed that span, for example, ‘Does the indicator signal a clear direction?’ to ‘Can needed data be accessed?’ and ‘What is the relevant reporting cycle?’ The listed considerations (table 3) based on the literature and views of panellists are a testament to the wide range of variables weighing on an indicator’s use that require thoughtful handling.

Third, an indicator’s fitness for purpose and fitness for use should be taken together to appraise actionability. For example, a policy-maker may identify a target to be measured in the scope of a strategy, yet for this specific purpose fitness for use considerations may not be met due to information system constraints or other contextual limitations. In another instance, an indicator may meet fitness for use considerations yet lack a clear and specific purpose and, in effect, misses a target audience. In both cases, the actionability of the indicator is compromised.

Lastly, as the expertise and lived experience of panellists served to highlight, the actionability of an indicator is not a guarantee of impact. Literature on the misuse, manipulation of data and unintended consequences of performance measurement depicts this.^45 73^ This distinction between action and impact underscores that while actionable healthcare performance indicators may be a precursor to better decision-making, the impact of an indicator weighs on considerations of its own.

### Applications and further research

This study has sought to consolidate the relevant literature and engage informants from differing contexts, areas of expertise and first-hand experiences for diverse insights. Future research should test the findings empirically, investigating purposes of use and fitness for use considerations by specific country contexts, governance structures, services delivery systems or areas of specialisation.

The findings of this study have a range of potential applications. In the context of the COVID-19 pandemic, actionable healthcare performance indicators have proven of paramount importance,^29 77^ and surges in publicly reported data illustrate the increased demand for information.^78 79^ The extent to which this information informs decision-making is a reflection of the alignment between an indicator’s intended purpose of use and related fitness for use considerations. The findings could also inform the selection of indicators for measurement frameworks and indicator sets that cascade healthcare system levels by priority areas (eg, tackling the misuse of antibiotic prescribing, strengthening integrated care), where different decision-making functions need to work in combination.

### Limitations

These findings may not be generalisable beyond the context of developed countries. The effect of system conditions, such as level of decentralisation, public–private mix and development status, has not been captured nor investigated given the targeted sample of informants, and as suggested should be explored empirically. The initial literature review was limited to English-language materials, which may also impact the generalisability of findings. Engaging expert panellists beyond English-speaking countries sought to minimise this. Some nuances may have become lost in choosing to summarise rather than transcribe interviews, although the advantages of our approach were found better suited for the study aims and design. In exploring performance indicators in the scope of healthcare, the study has not captured the broader use of indicators for public health despite its importance. Distributing panellists between panels was to the discretion of the study team for the purposes of the two-panel design, although many participants held positions or memberships suitable to both. The value of engaging panellists from different perspectives and stages took precedent. The prominence of panellists meant some were known to the authors. In order to avoid bias, a consistent interviewer was selected with the least previous engagement with panellists.

## Conclusion

Clarifying the meaning of actionable healthcare performance indicators is a perquisite to its further operationalisation. This study has explored the body of literature on the actionability of healthcare performance indicators for quality of care-related decision-making together with expert opinion and data user experiences in an effort to unpack the constructs of fitness for purpose and fitness for use. The study aimed to capture these constructs from a system perspective. The findings signal the importance of clarity and precision on an indicator’s purpose of use and context for the handling of methodological, contextual and managerial considerations weighing on its use in practice. Striking a better balance between the importance weighted to an indicator’s statistical merits and emphasis put to an indicator’s fitness for purpose and use is needed for indicators that are actionable for quality of care-related decision-making.

## Data Availability

Data are available upon reasonable request to the corresponding author.
