# Supplementary material for: Exploring the actionability of healthcare performance indicators for quality of care: a qualitative analysis of the literature, expert opinion and user experience
Source: BMJ Qual Saf. 2021 May 7;30(12):1010–20. doi: 10.1136/bmjqs-2020-011247 (PMC8606459; doi:10.1136/bmjqs-2020-011247)
Supplement: Supplementary data [file bmjqs-2020-011247supp001.pdf]

Online supplementary files

Appendix 1: Questions to expert panel

Summary of users and users of health care performance data

Through our exploratory literature review we have identified a range of data uses that transcend the levels of a health care system. This list is not exhaustive, nor does it intend to be. Importantly, it is also limited to the use of data for the purposes of clinical care rather than a broader population health perspective. Nonetheless, it does ambition to capture a minimum or core set of purposes for use and actors that resonate across different health care systems. Similarly, the listing of actors is not exhaustive and is to the exclusion of other actors that may carry out multiple functions. The classification has attempted to illustrate a unique user by each managerial function and actors that are explicitly assigned a mandate to improve quality of care and made directly accountable to a health care system. As such, actors such as professional and patient associations, academia and other research groups are excluded.

This listing of distinct uses has been distinguished at the micro- (clinical practice), meso- (institutions/organizations) and macro- (policy) level as shown by the nested sub-levels of the figure below and listed again in the table that follows for an editable version.

At each sub-level, a unique user can be identified – characterized as the primary actor with the responsible authority for a specific function (purpose of use). These purposes of use form different layers to decision-making; each embedded within one another. The interactions between layers signal the ways in which the users are related and co-dependent on the use and transfer of information, or as other authors have referred to as a 'data food chain', as some may reuse data collected and used by others.

- Is the list of purposes for use complete and accurate? If not, what is missing? Are there purposes for use that appear overlapping?
- Is the list of users complete and accurate? If not, what is missing?
- Is the cascading of users and uses accurate? If not, how can it be improved?

Table A1. Uses and users of health care performance indicators: listed

**Level**	**Managerial function (uses)**	**User**
Macro *Policy*	International comparisons	International organizations
	Governmental strategy	Government
	Governmental monitoring	Ministry of health
Meso *Institutional/ organizations*	Supervision/safety	Health care inspectorate
	Public reporting/accountability	Health care institute
	Purchasing/payment	Health care insurer
	Accreditation	Accreditation agency
Micro *Clinical practice*	Networks of professional performance	Management of institute/service provider network
	Operational quality management	Unit (department, ward) of service provider
	Quality improvement	Professional and professional team/pathways
	Care decision-making	Patient-professional

Other	…	…

Figure A1. Uses and users of health care performance indicators across the health system: visualized

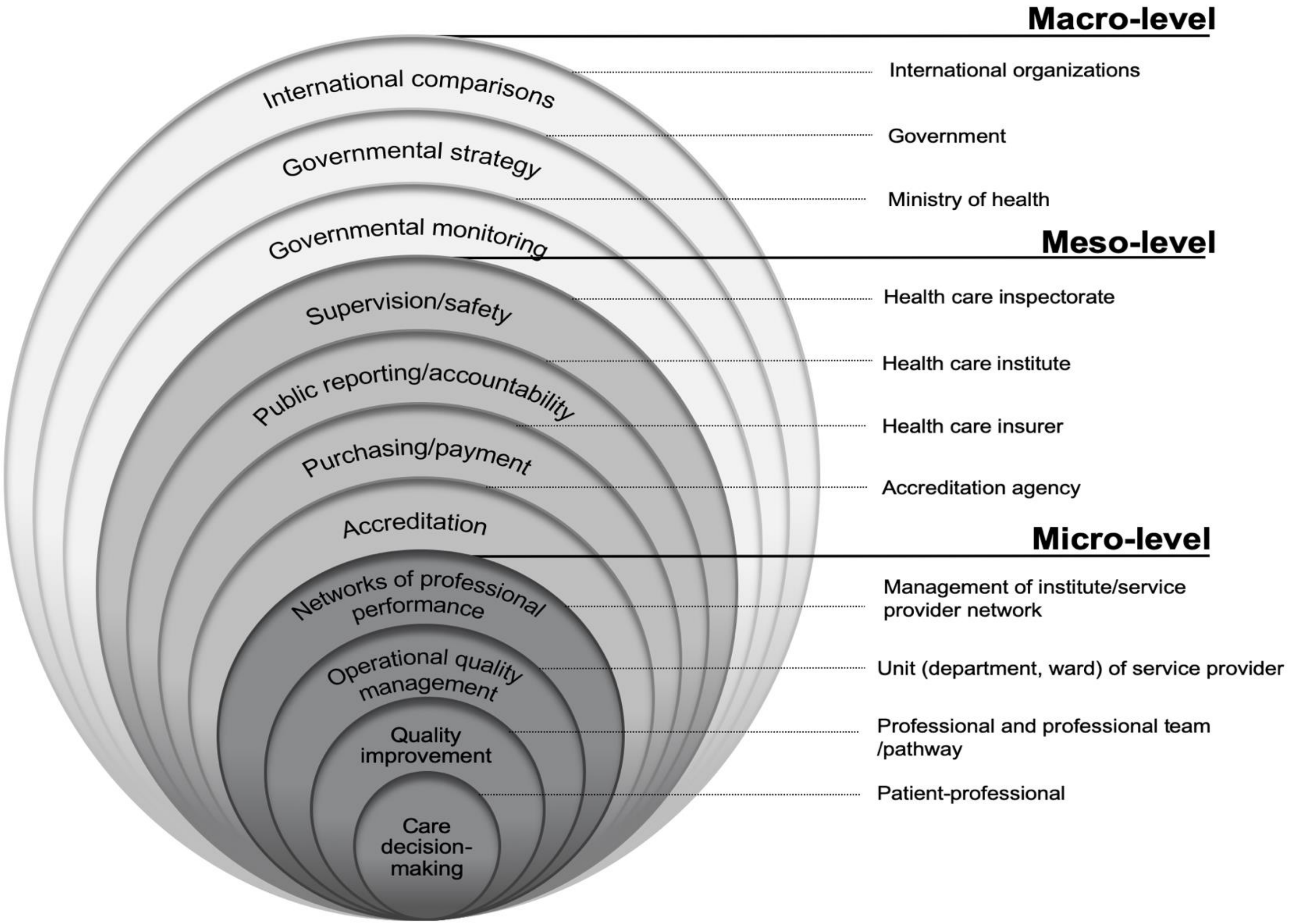

Methodological considerations

For each purpose for use, a range of methodological consequences (data requirements) can be anticipated. For example, the type of measure (e.g. more process measures at the micro-level vs more outcome measures at the macro-level) or confidence intervals (e.g. narrow for precision decisions of health practitioners vs wide for policy-makers). We have consolidated a range of methodologically relevant considerations (features of data) that has been highlighted in the literature as potentially relevant depending on the intended purpose of use. Examples to illustrate the variation for each of these considerations are listed in the table.

To further illustrate these considerations in practice, we have mapped existing indicators for measuring the performance of breast cancer services to different users and uses. The mapping serves to demonstrate the variability in information needs by user/use.

- Are the listed considerations meaningful? What is missed? What would you phrase differently? How can this list be expanded upon to serve its intended purpose of supporting users based on their intended purpose of use?

- How do you expect these considerations to vary based on specific uses and users from the table above?

Table A2. Fitness for use considerations and examples

Relevant considerations	Examples of variations
**Type of measure**	process, outcome, patient-reported
**Sources**	administrative, medical records or clinical, registries, patient
**Reference group**	unit or department, types of units, region, province, country etc.
**Reference period**	previous 1–6 quarters; 1–12 months; 1–6 years; previous year, etc.
**Comparators**	past performance, targets, reference group
**Compounds (composites)**	selection of indicators, weights
**Calculation of values**	standard deviation, means, median, other percentiles (75th), rank, top 10% mean (ABC method), individual peer scores, ranges
**Statistical chart options**	funnel plot, Shewhart chart, scan statistics, moving average, sets method
**Types of analysis**	benchmarking, time trend, international comparison
**Confidence intervals**	narrow for precision decisions (practitioners) vs wide for policy-makers
**Risk adjustments**	variable specification (demographic, clinical factors, socio-economic, health related, patient preferences), source, weighting scheme
**Others…**	…

General reflections

- Do you agree with the construction and approach to exploring fitness for use of health care performance data as pursued in this study?
- Are you aware of any existing studies that should be consulted in the scope of this work?

Appendix 2: Questions to user panel

Purposes of use of health care performance indicators

Uses of health care performance indicators can be differentiated at the micro- (clinical practice), meso- (institutions/organizations) and macro- (policy) level of health systems. Methodologically relevant distinctions by the purposes for using health care performance indicators are shown in the nested sub-levels of the figure below. For example, at the micro-level, uses of health care performance indicators include for informed choice (e.g. by patients), individual professional performance improvement and practice or team performance improvement. Cross-cutting these uses are factors that ultimately weigh on the extent to which a specific purpose of use can be met. These feed-in factors can be clustered around the specific country context, governance and inputs (e.g. research, data infrastructure).

1. Based on the figure (A2), how would you describe your most predominate purpose for using health care performance indicators?

2. For this purpose, what in your opinion constitutes a good indicator? That is, what information do you need? Can you give examples?

3. Can you describe how you make use of health care performance indicators? Who is your target end-user? What specific strategies or mechanisms are relied on (e.g. public reporting, licensing or accreditation, financial incentives, etc.)?

Figure A2. Uses and users of health care performance indicators across the health system: visualize

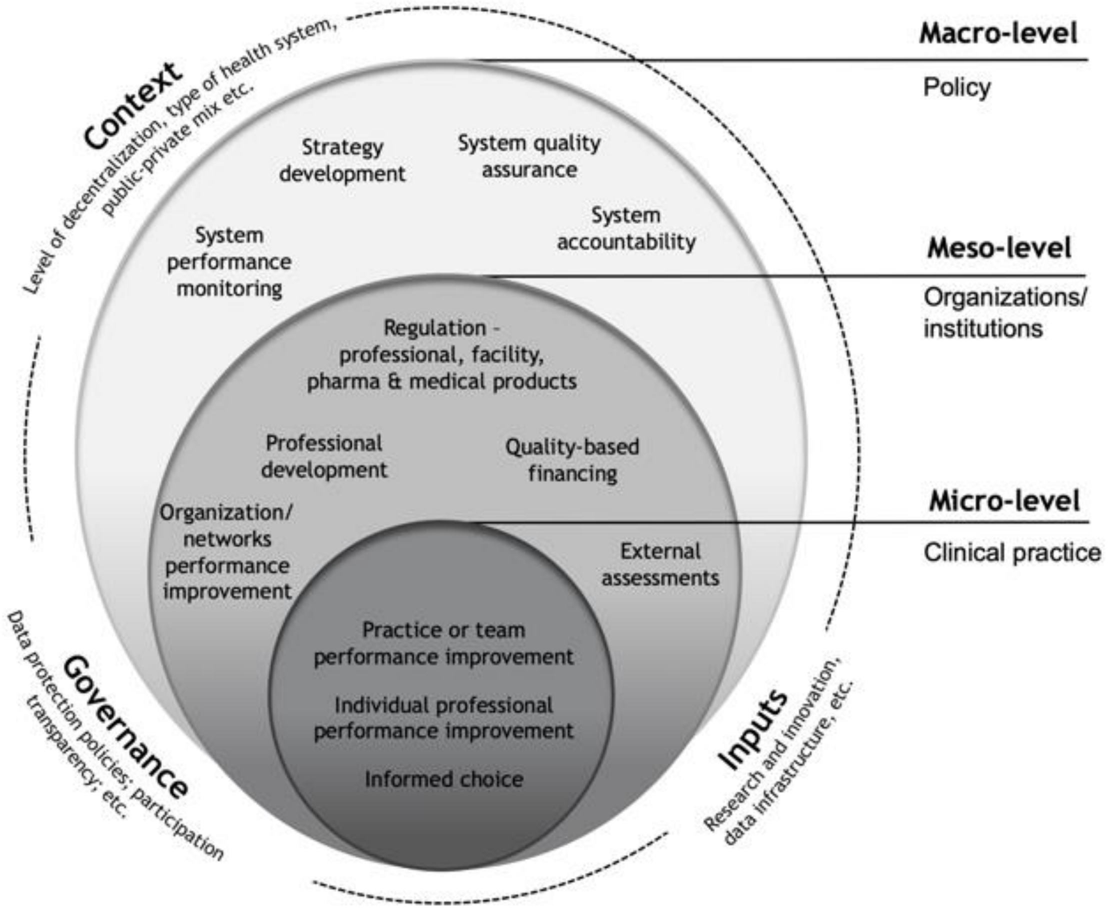

Considerations for the use of health care performance indicators

We recognize that depending on the purpose of use, each user has different information needs. The type of indicator (e.g. process, structure outcome), sources of data (e.g. administrative, clinical, survey data), timeliness (e.g. weekly, quarterly, annually) or type of analysis (e.g. benchmarking, time trend, international comparison), are among some of these differences.

Given this variability, there are a range of both practical and methodological considerations that may potentially affect the use of health care performance indicators. These considerations cannot be reduced solely to the analysis of indicators. For example, if an indicator is well-defined, collected and analyzed but is not presented optimally, what it signals may not be clear to the intended user for decision-making purposes. In other instances, it may be the data itself that is the main issue, because it cannot be linked, is of poor quality or incomplete. We have clustered five key factors and potentially relevant considerations depending on the intended purpose of use of an indicator that have been highlighted in the literature and by the first round of panelists.

1. Based on your use of health care performance indicators, what are your critical needs across the indicator cycle shown in Fig.A3? That is, for each stage in the cycle as shown, what would make the ideal conditions for your purposes?

2. Which stage in your opinion is most important for the actionability of an indicator?

3. Is the information currently used by your organization or practice useful for your purposes? That is, are you able to make decisions and learn from the information? How could it be better?

4. In your opinion, what are the main obstacles users face to make health care performance data actionable?

Figure A3. Use cycle of health care performance indicators

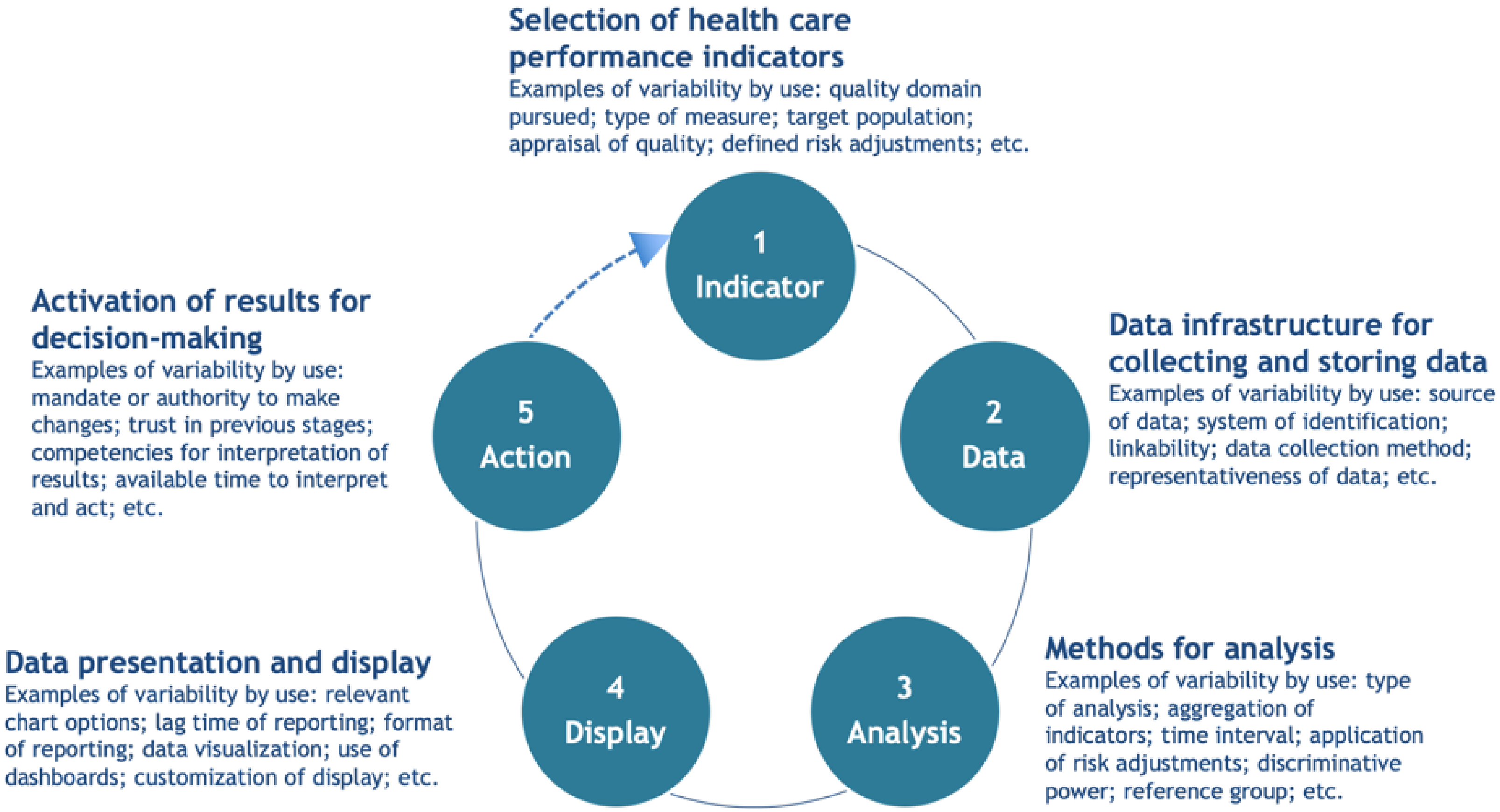

BMJ Publishing Group Limited (BMJ) disclaims all liability and responsibility arising from any reliance placed on this supplemental material which has been supplied by the author(s)

Appendix 3: Panelists

**Expert panelists**				**User panelists**			
**#**	**Expertise** (key words)	**Literature consulted**	**Country**	**#**	**Organization type**	**Level**	**Country**
1	Quality of care	[1-6]	Australia	1	Standards	Macro	United States
2	Performance measurement	[7-10]	United States	2	Governmental	Macro	Belgium
3	Performance measurement	[11-16]	Germany	3	Research/academia	Micro	United Kingdom
4	Quality of care	[1,2,5,6,17]	Australia	4	Standards	Meso	Canada
5	Governance	[18-20]	Canada	5	Governmental	Macro	Ireland
6	Performance measurement	[21-23]	Denmark	6	Governmental	Macro	Canada
7	Data/information	[24-26]	Netherlands	7	Health services	Meso	Germany
8	Management	[27-29]	Italy	8	Improvement	Macro	United States
9	Data/information systems	[30-32]	Canada	9	Governmental	Macro	Canada
10	Performance measurement	[33-36]	United Kingdom	10	Health services	Micro	Netherlands
11	Management	[37-39]	Netherlands	11	Research/academia	Meso	Netherlands
12	Quality of care	[40-42]	United States	12	Health services	Meso	United States
13	Performance measurement	[33,35,36,43]	United Kingdom	13	Standards	Meso	Netherlands
14	Governance	[44-46]	Denmark	14	Health services	Micro	Netherlands
15	Governance	[23,47,48]	Canada	15	Improvement	Macro	Netherlands
16	Data/information systems	[49-51]	Netherlands	16	Governmental	Macro	Canada

Appendix references

1. Braithwaite J, Hibbert P, Blakely B, Plumb J, Hannaford N, Long JC, et al. Health system frameworks and performance indicators in eight countries: A comparative international analysis. SAGE Open Medicine. 2017;5:2050312116686516.
2. Braithwaite J, Hibbert PD, Jaffe A, White L, Cowell CT, Harris MF, et al. Quality of Health Care for Children in Australia, 2012-2013. Jama. 2018;319(11):1113-24.
3. Mannion R, Braithwaite J. Unintended consequences of performance measurement in healthcare: 20 salutary lessons from the English National Health Service. Intern Med J. 2012;42(5):569-74.
4. Mumford V, Greenfield D, Hogden A, Debono D, Forde K, Westbrook J, et al. Development and application of an indicator assessment tool for measuring health services accreditation programs. BMC Res Notes. 2015;8:363.
5. Hibbert PD, Wiles LK, Cameron ID, Kitson A, Reed RL, Georgiou A, et al. CareTrack Aged: the appropriateness of care delivered to Australians living in residential aged care facilities: a study protocol. BMJ Open. 2019;9(6):e030988.
6. Runciman WB, Hunt TD, Hannaford NA, Hibbert PD, Westbrook JI, Coiera EW, et al. CareTrack: assessing the appropriateness of health care delivery in Australia. Med J Aust. 2012;197(2):100-5.
7. Damberg CL, Sorbero ME, Lovejoy SL, Lauderdale K, Wertheimer S, Smith A, et al. An Evaluation of the Use of Performance Measures in Health Care. Rand health quarterly. 2012;1(4):3-.
8. Damberg CL, Baker DW. Improving the Quality of Quality Measurement. Journal of general internal medicine. 2016;31 Suppl 1(Suppl 1):8-9.
9. Friedberg MW, Damberg CL. A Five-Point Checklist To Help Performance Reports Incentivize Improvement And Effectively Guide Patients. Health Affairs. 2012;31(3):612-8.
10. Parast L, Doyle B, Damberg CL, Shetty K, Ganz DA, Wenger NS, et al. Challenges in Assessing the Process–Outcome Link in Practice. Journal of general internal medicine. 2015;30(3):359-64.
11. Shaw C, Groene O, Berger E. Chapter 8: External institutional strategies: accreditation, certification, supervision. In: Busse R, Klazinga N, Panteli D, Quentin W, editors. Improving healthcare quality in Europe: characteristics, effectiveness and implementation of different strategies Copenhagen WHO Regional Office for Europe 2019.
12. Groene O. Improving the capacity for learning and improvement in health care. International Journal for Quality in Health Care. 2018;30(3):159-60.
13. Groene O, Sunol R. Patient involvement in quality management: rationale and current status. J Health Organ Manag. 2015;29(5):556-69.
14. Taylor A, Neuburger J, Walker K, Cromwell D, Groene O. How is feedback from national clinical audits used? Views from English National Health Service trust audit leads. Journal of health services research & policy. 2016;21(2):91-100.
15. Wagner C, Groene O, Thompson CA, Dersarkissian M, Klazinga NS, Arah OA, et al. DUQuE quality management measures: associations between quality management at hospital and pathway levels. International journal for quality in health care : journal of the International Society for Quality in Health Care. 2014;26 Suppl 1(Suppl 1):66-73.
16. Hilarion P, Suñol R, Groene O, Vallejo P, Herrera E, Saura RM. Making performance indicators work: the experience of using consensus indicators for external assessment of health and social services at regional level in Spain. Health Policy. 2009;90(1):94-103.
17. Hibbert Pea. Performance indicators used internationally to report publicly on healthcare organisations and local health systems. South Wales: Australian Institute of Health Innovation: University of New South Wales; 2013.
18. Lavis JN. Finding and using research evidence: Summary sheet. Hamilton, Canada: McMaster Health Forum 2019.
19. Fretheim A, Oxman AD, Lavis JN, Lewin S. SUPPORT Tools for Evidence-informed Policymaking in health 18: Planning monitoring and evaluation of policies. Health research policy and systems. 2009;7(1):S18.

20. Lavis JN, Hammill AC. Governance arrangements. In: JN L, editor. Ontario's health system: Key insights for engaged citizens, professionals and policymakers. Hamilton: McMaster Health Forum 2016.
21. Mainz J. Defining and classifying clinical indicators for quality improvement. International Journal for Quality in Health Care. 2003;15(6):523-30.
22. Mainz J. Developing evidence-based clinical indicators: a state of the art methods primer. International Journal for Quality in Health Care. 2003;15(suppl_1):i5-i11.
23. Carinci F, Van Gool K, Mainz J, Veillard J, Pichora EC, Januel JM, et al. Towards actionable international comparisons of health system performance: expert revision of the OECD framework and quality indicators. International Journal for Quality in Health Care. 2015;27(2):137-46.
24. Medlock S, Wyatt JC. Health Behaviour Theory in Health Informatics: Support for Positive Change. Studies in health technology and informatics. 2019;263:146-58.
25. Medlock S, Eslami S, Askari M, Arts DL, van de Glind EM, Brouwer HJ, et al. For which clinical rules do doctors want decision support, and why? A survey of Dutch general practitioners. Health Informatics J. 2019;25(3):1076-90.
26. Askari M, Wierenga PC, Eslami S, Medlock S, De Rooij SE, Abu-Hanna A. Studies pertaining to the ACOVE quality criteria: a systematic review. International journal for quality in health care : journal of the International Society for Quality in Health Care. 2012;24(1):80-7.
27. Nuti S. Let's play the patients music: A new generation of performance measurement systems in healthcare. Management Decision. 2018;56(10):2252-72.
28. Nuti S, De Rosis S, Bonciani M, Murante AM. Rethinking Healthcare Performance Evaluation Systems towards the People-Centredness Approach: Their Pathways, their Experience, their Evaluation. Healthc Pap. 2017;17(2):56-64.
29. Nuti S, Bini B, Ruggieri TG, Piaggesi A, Ricci L. Bridging the Gap between Theory and Practice in Integrated Care: The Case of the Diabetic Foot Pathway in Tuscany. International journal of integrated care. 2016;16(2):9-.
30. OECD. Health in the 21st Century: putting data to work for stronger health systems. Paris: OECD; 2019.
31. OECD. Health data governance: privacy, monitoring and research - policy brief Paris: OECD; 2015.
32. OECD. Strengthening health information infrastructure for health care quality governance: good practices, new opportunities and data privacy challenges Paris: OECD 2013.
33. Smith P, Mossialos E, Papanicolas I. Performance measurement for health system improvement: eperiences, challenges and prospects. Copenhagen: WHO Regional Office for Europe; 2008.
34. Papanicolas I, Jha AK. Challenges in International Comparison of Health Care Systems. JAMA. 2017;318(6):515-6.
35. Smith P, Mossialos E, Papanicolas I, Leatherman S. Principles of performance measurement In: Smith P, Mossialos E, Papanicolas I, Leatherman S, editors. Performance measurement for health system improvement Cambridge: Cambridge University Press; 2009.
36. Papanicolas I, Smith PC. Health system performance comparison: An agenda for policy, information and research Maidenhead: McGraw-Hill 2013.
37. Rotar AM, van den Berg MJ, Kringos DS, Klazinga NS. Reporting and use of the OECD Health Care Quality Indicators at national and regional level in 15 countries. International journal for quality in health care : journal of the International Society for Quality in Health Care. 2016;28(3):398-404.
38. Rotar AM, van den Berg MJ, Klazinga NS. An expert-based mapping of healthcare system strategies to support rational drug prescribing in primary care across 13 European countries. Health research policy and systems. 2020;18(1):102.
39. Rotar AM, Botje D, Klazinga NS, Lombarts KM, Groene O, Sunol R, et al. The involvement of medical doctors in hospital governance and implications for quality management: a quick scan in 19 and an in depth study in 7 OECD countries. BMC Health Serv Res. 2016;16 Suppl 2(Suppl 2):160.
40. Van der Wees PJ, Nijhuis-van der Sanden MWG, van Ginneken E, Ayanian JZ, Schneider EC, Westert GP. Governing healthcare through performance measurement in Massachusetts and the Netherlands. Health Policy. 2014;116(1):18-26.

41. Van Der Wees PJ, Nijhuis-Van Der Sanden MWG, Ayanian JZ, Black N, Westert GP, Schneider EC. Integrating the Use of Patient-Reported Outcomes for Both Clinical Practice and Performance Measurement: Views of Experts from 3 Countries. The Milbank quarterly. 2014;92(4):754-75.
42. Geissler KH, Friedberg MW, SteelFisher GK, Schneider EC. Motivators and barriers to using patient experience reports for performance improvement. Med Care Res Rev. 2013;70(6):621-35.
43. Smith PC, Anell A, Busse R, Crivelli L, Healy J, Lindahl AK, et al. Leadership and governance in seven developed health systems. Health Policy. 2012;106(1):37-49.
44. Barbazza E, Tello J. A review of health governance: Definitions, dimensions and tools to govern Health Policy 2014;116:1-11.
45. Tello JE, Barbazza E, Waddell K. Review of 128 quality of care mechanisms: A framework and mapping for health system stewards. Health Policy. 2020;124(1):12-24.
46. Tello J, Baez-Camargo C. Strengthening health system accountability: a WHO European Region multi-country study. Copenhagen WHO Regional Office for Europe 2015.
47. Veillard JH, Brown AD, Baris E, Permanand G, Klazinga NS. Health system stewardship of National Health Ministries in the WHO European region: concepts, functions and assessment framework. Health Policy. 2011;103(2-3):191-9.
48. Veillard J, Champagne F, Klazinga N, Kazandjian V, Arah OA, Guisset AL. A performance assessment framework for hospitals: the WHO regional office for Europe PATH project. International journal for quality in health care : journal of the International Society for Quality in Health Care. 2005;17(6):487-96.
49. Verheij RA, Curcin V, Delaney B, McGilchrist MM. Possible sources of bias in primary care electronic health record data use and reuse Journal of medical internet research. 2018;20(5).
50. Verheij R, Witvliet C, Jansen T, Hooiveld M, Hilten O van. Reuse of routine care data for policy and science: how things can be improved [Dutch]. Utrecht: Nivel; 2019.
51. van der Bij S, de Hoon S, Nielen M, de Jong A, de Boer D, Verheij R. Routine recorded care data as a source of quality information [Dutch]. Netherlands Nivel; 2016.
